# Introducing dual practice may threaten equitable surgical access in Canada

**DOI:** 10.1016/j.lana.2026.101442

**Published:** 2026-03-09

**Authors:** Babatope O. Adebiyi

**Affiliations:** Section of Rheumatology, Department of Pediatrics, Cumming School of Medicine, University of Calgary, 2500 University Drive NW, Calgary, T2N 1N4, Alberta, Canada

Alberta's proposal to permit surgeons to perform both publicly funded and privately paid procedures represents a departure from Canada's commitment to universal, publicly financed health care.^1^ Although presented as a strategy to expand surgical capacity and reduce wait times, this shift introduces structural incentives that could undermine equitable access to medically necessary procedures, particularly for low-income, rural, and Indigenous populations who already face barriers to timely care.^2^

Global experiences from Ireland, Australia, and the United Kingdom show that when surgeons work in both public and private systems, clinicians reduce their availability in public hospitals in favour of private activity; wait times lengthen for publicly funded patients and private queues shorten for those able to pay.^3^, ^4^, ^5^ These dynamics risk widening inequities in surgical access.

Similar patterns have emerged in the Americas. In Chile, mixed public–private arrangements have been associated with longer waits for orthopaedic surgery among publicly insured patients than among those covered by private insurance.^6^ In Brazil, where dual practice is widespread and most physicians work in both sectors, diversion of surgical activity to private facilities has weakened public capacity, leaving poorer patients facing longer waits despite universal coverage.^7^

Canadian data illustrate how fragile surgical access already is. In 2024, 68% of hip and 61% of knee replacements were completed within the recommended 26-week benchmark nationally, below pre-pandemic levels.^8^ In Alberta, only 59% of hip and 49% of knee replacements met the 26-week benchmark in 2023.^8^ Diverting scarce surgical labour toward private facilities through dual practice could slow progress on reducing public wait times.

Introducing dual practice at a time when health systems are strained by workforce shortages may erode public-sector capacity. In provinces with large rural populations, even small drops in public-sector availability can cause delays, poorer outcomes, and widening disparities.^2^ Indigenous communities could be disproportionately disadvantaged by a system that enables faster pathways for those with financial means.^2^ Reducing surgical backlogs is legitimate, but solutions should strengthen, not fragment, the public system. Evidence-informed alternatives include expanding operating-room hours, improving perioperative efficiency, and addressing staffing.

Alberta's dual-practice proposal should therefore be approached with caution. Without safeguards, such as limits on private caseloads, transparent reporting of wait times, and protections for underserved populations, the model risks entrenching inequities rather than alleviating them. Policymakers must ensure that changes reinforce the fundamental value that has long distinguished Canada's health system: fair access to essential surgery based on need, not ability to pay.

## Declaration of interests

The author declares no competing interests.
